# Sodium‐glucose cotransporter 2 inhibitors and outcomes in transthyretin amyloid cardiomyopathy: Systematic review and meta‐analysis

**DOI:** 10.1111/eci.14392

**Published:** 2025-01-27

**Authors:** Paschalis Karakasis, Panagiotis Theofilis, Dimitrios Patoulias, Art Schuermans, Panayotis K Vlachakis, Aleksandra Klisic, Manfredi Rizzo, Nikolaos Fragakis

**Affiliations:** ^1^ Second Department of Cardiology, Hippokration General Hospital Aristotle University of Thessaloniki Thessaloniki Greece; ^2^ First Cardiology Department, School of Medicine, Hippokration General Hospital National and Kapodistrian University of Athens Athens Greece; ^3^ Second Propedeutic Department of Internal Medicine, Faculty of Medicine, School of Health Sciences Aristotle University of Thessaloniki Thessaloniki Greece; ^4^ Cardiovascular Research Center and Center for Genomic Medicine Massachusetts General Hospital Boston Massachusetts USA; ^5^ Faculty of Medicine KU Leuven Leuven Belgium; ^6^ Primary Health Care Center, Faculty of Medicine University of Montenegro Podgorica Montenegro; ^7^ Ras Al Khaimah Medical and Health Sciences University Ras Al Khaimah United Arab Emirates; ^8^ School of Medicine, Department of Health Promotion, Mother and Child Care, Internal Medicine and Medical Specialties (Promise) University of Palermo Palermo Italy

**Keywords:** arrhythmias, ATTR‐CM, hospitalization, mortality, SGLT2 inhibitors, transthyretin amyloid cardiomyopathy

## Abstract

**Background:**

Transthyretin amyloid cardiomyopathy (ATTR‐CM) commonly leads to heart failure but has traditionally been an exclusion criterion in randomized clinical trials (RCTs) of sodium‐glucose cotransporter 2 inhibitors (SGLT2i); therefore, the effects of these drugs in this population remain undocumented. In light of recent studies, this meta‐analysis aimed to investigate the effect of SGLT2i on the prognosis of patients with ATTR‐CM.

**Methods:**

A comprehensive search of Medline, Scopus, and the Cochrane Library was conducted up to November 17, 2024. Study selection, data extraction and quality assessment were carried out independently by two investigators. Associations of SGLT2i with outcomes were pooled using random‐effects meta‐analyses.

**Results:**

A total of five studies (9766 participants, 4 propensity score‐matched) were included. The use of SGLT2i was associated with significant reductions in all‐cause mortality [hazard ratio (HR) .54, 95% confidence interval (CI) .44–.66], cardiovascular mortality (HR .39, 95% CI .23–.65), major adverse cardiovascular events (HR .71, 95% CI .61–.83), and heart failure hospitalizations (HFHs) (HR .63, 95% CI .52–.77) compared to non‐use. The odds of cardiac arrhythmias were significantly lower among SGLT2i users compared to non‐users [odds ratio (OR) .73, 95% CI .65–.83]. Specifically, SGLT2i use was associated with significant reductions in the odds of atrial fibrillation (AF) (OR .75, 95% CI .62–.91), ventricular tachycardia (OR .72, 95% CI .59–.88), and sudden cardiac arrest (OR .71, 95% CI .50–.99).

**Conclusions:**

The use of SGLT2is may be associated with a more favourable prognosis in patients with ATTR‐CM. Adequately powered, long‐term RCTs are required to validate the available observational evidence.

## Graphical abstract

Patients with transthyretin amyloid cardiomyopathy (ATTR‐CM) were excluded from randomized clinical trials (RCTs) evaluating sodium‐glucose cotransporter 2 inhibitors (SGLT2i); as a result, the effects of these agents on this population remain underexplored. The present meta‐analysis suggests that the use of SGLT2i may be associated with reduced all‐cause mortality, cardiovascular mortality, major adverse cardiovascular events, hospitalizations for heart failure and cardiac arrhythmias compared to non‐use in patients with ATTR‐CM. Long‐term RCTs are required to validate the existing observational evidence.

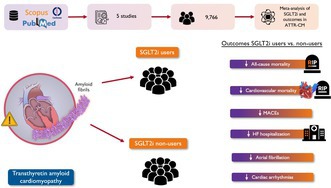

**Key points**

- Patients with transthyretin amyloid cardiomyopathy (ATTR‐CM) were excluded from randomized clinical trials (RCTs) evaluating sodium‐glucose cotransporter 2 inhibitors (SGLT2i); as a result, the effects of these agents on this population remain underexplored.
- This meta‐analysis suggests that the use of SGLT2i may be associated with reduced all‐cause mortality, cardiovascular mortality, major adverse cardiovascular events, hospitalizations for heart failure and cardiac arrhythmias compared to non‐use in patients with ATTR‐CM.
- Long‐term RCTs are required to validate the existing observational evidence.

## 1 INTRODUCTION

Transthyretin amyloid cardiomyopathy (ATTR‐CM) results from the misfolding and aggregation of transthyretin, a plasma protein, into amyloid fibrils that deposit in the myocardial extracellular matrix.^1^ This accumulation ultimately leads to a progressive impairment of cardiac function.^1^ ATTR‐CM can be inherited as an autosomal dominant trait due to pathogenic variants in *TTR* (i.e., the transthyretin gene; “ATTRv‐CM”) or due to accumulation of wild‐type transthyretin (“ATTRwt‐CM”).^1^ Historically, ATTR‐CM was considered a rare condition, but the advancements in noninvasive diagnostic modalities has revealed a higher prevalence than previously recognized.^2^, ^3^, ^4^ These advances have also catalysed the development of several promising therapies aimed at modifying the disease course.^2^, ^3^, ^4^

While several treatments for ATTR‐CM are under development, such as antisense oligonucleotides, silencing RNA and CRISPR‐based strategies,^5^ tafamidis and acoramidis are currently the only pharmacological agents approved for its treatment.^6^, ^7^, ^8^ Tafamidis functions by selectively binding to and stabilizing circulating transthyretin in its native soluble conformation, thereby reducing its propensity to misfold and aggregate into amyloid fibrils.^6^, ^7^ Similarly, acoramidis is a high‐affinity transthyretin stabilizer that inhibits the dissociation of tetrameric transthyretin, leading to over 90% stabilization across the dosing interval.^8^ Evidence from the phase 3 ATTR‐ACT trial (Tafamidis in Transthyretin Cardiomyopathy Clinical Trial) demonstrated that tafamidis significantly reduced cardiovascular‐related hospitalizations and mortality rates.^9^ However, its widespread adoption has been hindered by its prohibitive cost, limiting accessibility in many regions.^10^ Consequently, supportive care remains the cornerstone of management. The utility of conventional heart failure (HF) therapies in ATTR‐CM has been contentious, largely due to the exclusion of amyloid patients from traditional HF clinical trials.^11^ Nevertheless, a large retrospective analysis of ATTR‐CM patients revealed lower all‐cause mortality among those receiving beta blockers (in cases of ejection fraction <40%) and mineralocorticoid receptor antagonists (MRAs), highlighting a critical need for further investigation into the efficacy of standard HF treatments in this challenging patient population.^11^

Sodium‐glucose cotransporter 2 inhibitors (SGLT2i) have emerged as a class of cardio‐renal‐metabolic therapies, demonstrating significant benefits such as reducing hospitalizations for HF, enhancing cardiac function and slowing the progression of chronic kidney disease.^12^, ^13^, ^14^, ^15^, ^16^ Despite these established advantages in broader cardiovascular and renal populations, their efficacy in ATTR‐CM remains insufficiently defined. Preliminary investigations suggest that SGLT2i are well‐tolerated in this patient cohort^17^, ^18^; however, robust evidence regarding their therapeutic impact is lacking. Accordingly, this meta‐analysis sought to systematically evaluate the impact of SGLT2i on clinical outcomes in individuals diagnosed with ATTR‐CM.

## 2 MATERIALS AND METHODS

The study adhered to the guidelines specified in the Cochrane Handbook for Systematic Reviews,^19^ and was reported following the PRISMA 2020 standards for systematic reviews and meta‐analyses^20^ (Table S1). The study protocol was registered a priori on the Open Science Network (10.17605/OSF.IO/5FT42), and no amendments were made to the original protocol thereafter.

### 2.1 Search strategy

A comprehensive literature search was systematically conducted by two independent researchers across multiple databases, including MEDLINE (via PubMed), Scopus, and the Cochrane Central Register of Controlled Trials, covering all records from database inception through November 17, 2024. No limitations were applied concerning date, language, publication status, or year at the initial search stage. The primary search terms included “sglt2 inhibitor”, “all‐cause mortality”, “cardiovascular mortality”, “adverse cardiovascular events”, “hospitalization” and “arrhythmias” applied both as free‐text keywords and using Medical Subject Headings (MeSH). Additional searches were carried out manually on ClinicalTrials.gov, the Epistemonikos database, and Google Scholar. Furthermore, backward and forward citation tracking was employed using the *citationchaser* package in R.^21^ Detailed search strategies are provided in Tables S2–S4.

### 2.2 Eligibility criteria

#### 2.2.1 Inclusion criteria

Eligible studies included randomized controlled trials (RCTs) or observational studies that examined the effects of SGLT2i on clinical outcomes in adults (aged 18 years or older) with ATTR‐CM.

#### 2.2.2 Exclusion criteria

Studies with the following characteristics were excluded: (i) case reports, case series and narrative reviews; (ii) editorials, letters, commentaries and expert opinions; (iii) clinical practice guidelines, protocols and dissertations; (iv) studies reporting exclusively on laboratory markers or other surrogate outcomes; and (v) studies for which the full text was not retrievable.

### 2.3 Outcomes

The outcomes for meta‐analysis included all‐cause mortality, cardiovascular mortality, major adverse cardiovascular events (MACE), heart failure hospitalizations (HHFs), and cardiac arrhythmias—including atrial fibrillation (AF), ventricular tachycardia (VT), and sudden cardiac arrest (SCA)—comparing SGLT2i users with non‐users.

### 2.4 Study Selection

In the initial phase, two authors independently screened all titles and abstracts from records identified through the prespecified search strategy. To enhance the sensitivity of the study selection process, discrepancies at this stage did not result in exclusions. Subsequently, two investigators independently evaluated the full texts of potentially eligible studies. Any disagreements were resolved through consensus or, if necessary, by consulting a senior author. The screening process in the initial phase was facilitated by the Abstrackr tool,^22^ while Mendeley was utilized for reference management.

### 2.5 Data extraction

A data extraction form was developed and underwent a pilot phase using a subset of four studies. Following a series of training and calibration sessions, a standardized form for data extraction was finalized. Data extraction was conducted independently in duplicate, with any discrepancies resolved by consensus or, when necessary, by consulting a senior author. For each study, we extracted information on sample size, relevant clinical and demographic characteristics, and adjusted effect estimates for outcomes of interest, when such adjustments were available. Additionally, corresponding authors from the included studies were contacted to request supplementary information if data were missing or not explicitly provided in the published reports.

### 2.6 Quality assessment

Two authors independently evaluated the quality of the identified studies using the Risk Of Bias In Non‐randomized Studies—of Interventions (ROBINS‐I) tool, which provides a systematic framework for assessing the risk of bias in observational epidemiological studies.^23^ Any discrepancies during the assessment were resolved through discussion or, if necessary, by consulting a third author.

### 2.7 Data analysis

All analyses were conducted using R Statistical Software, version 4.2, utilizing the *meta*, package. Categorical variables are reported as frequencies and percentages (%), while continuous variables are presented as means with standard deviations (SDs) for normally distributed data, and as medians with interquartile ranges (IQR) for non‐normally distributed data. To estimate the relative effects between intervention and control groups, effect estimates and their 95% confidence intervals (CIs) were pooled using random‐effects pairwise models, employing the restricted maximum likelihood estimator for between‐study variance within a frequentist framework. Since Jaiswal et al.^24^ did not provide hazard ratios (HRs) estimates, binomial data were converted to HR using established methods.^25^ A two‐tailed *p*‐value of less than .05 was considered statistically significant for summary effect estimates. All analyses followed an intention‐to‐treat approach.

The *I* ^2^ statistic was calculated to assess the proportion of total variability due to between‐study heterogeneity, indicating the degree of inconsistency across studies. Additionally, heterogeneity was formally assessed using Cochran's Q test. Generally, thresholds of 25%, 50% and 75% are used to indicate low, moderate and high heterogeneity, respectively.^26^ To evaluate small‐study effects and potential publication bias, contour‐enhanced funnel plots (plotting effect size against standard error) were used for visual assessment, with Egger's test providing a formal statistical evaluation.

## 3 RESULTS

### 3.1 Study selection and characteristics

**FIGURE 1:**
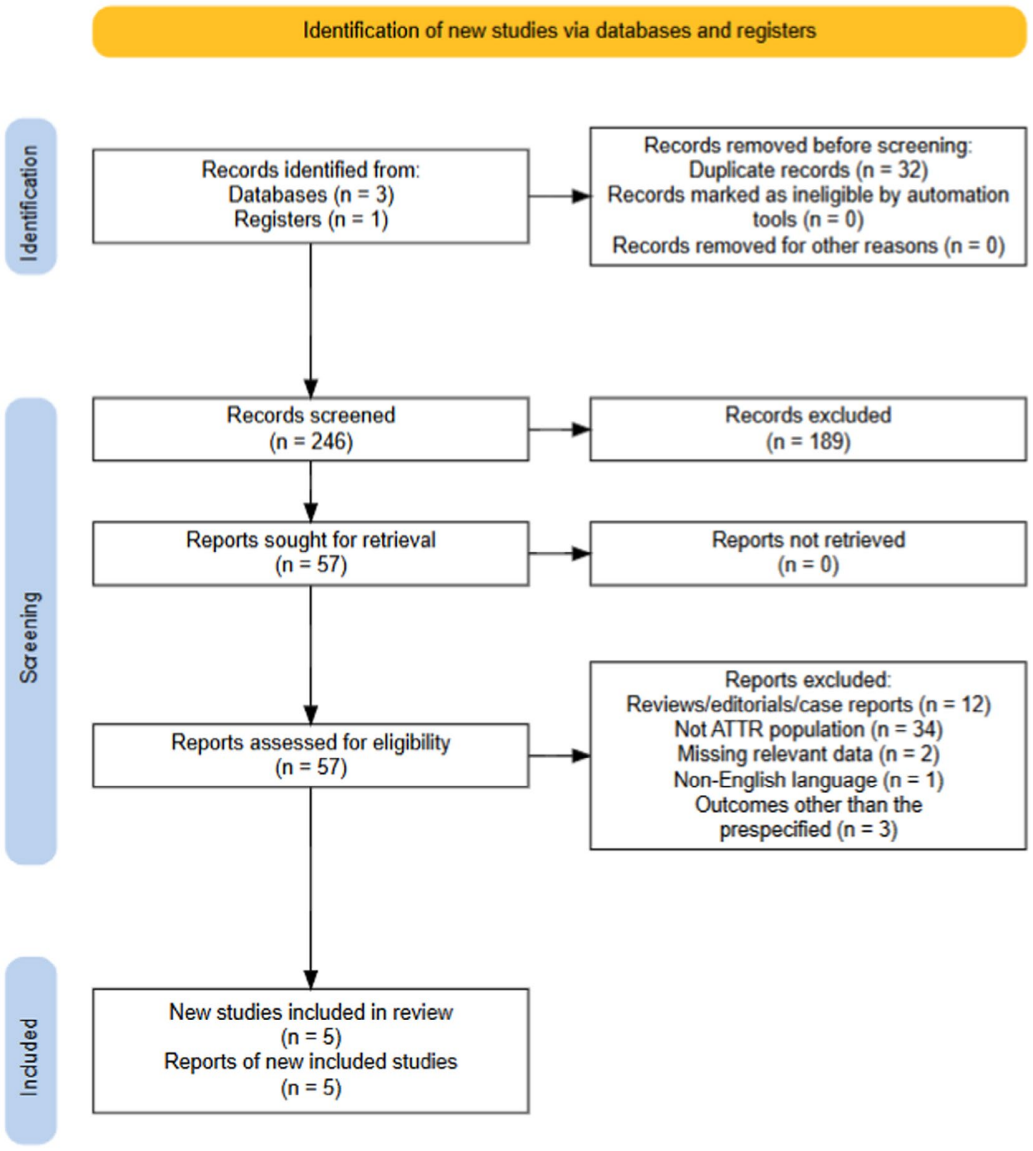
Preferred reporting items for systematic reviews and meta‐analyses (PRISMA) flow diagram.

The PRISMA diagram detailing the database search and study selection process is provided in Figure 1. After duplicate records were removed, an initial set of 246 identified studies was screened by title and abstract. Of these, 189 studies were excluded. The remaining 57 studies underwent a thorough full‐text evaluation, resulting in the inclusion of 5 studies that satisfied the eligibility criteria.^24^, ^27^, ^28^, ^29^, ^30^

In total, five studies (4 of which were propensity score‐matched), involving 9766 participants, were analysed. The median age was 77 (IQR: 76–79) years, and the median proportion of male participants was 81% (IQR: 76–89). The median left ventricular ejection fraction (LVEF) was 50% (IQR: 47–51). All studies accounted for disease‐modifying therapy (tafamidis or patisiran), with adjustments made either at the propensity score‐matching stage^24^, ^27^, ^29^, ^30^ or subsequently through regression.^28^ The baseline characteristics of included studies are presented in Table 1. The full list of variables used for confounder adjustment in each primary study is detailed in Table S5.

**TABLE 1:** Baseline characteristics of included studies.

Study, year	Follow‐up (years)	Mean age (years)		Male sex (%)		LVEF (%)		Diabetes (%)		NYHA functional class II (%)		Loop diuretic agents (%)		Disease‐modifying therapy (%)	
		S	C	S	C	S	C	S	C	S	C	S	C	S	C
Jaiswal et al. 2024^24^	3	74.2	74.4	64.7	65.1	50.5	51.5	53.4	54.4	2.5^a^	2.5^a^	87.3	90	13.1	11.2
Porcari et al. 2024^27^	1	77.2	76.7	90.5	89.1	45.8	46	89	94	65.9	66.8	84.5	84.1	20.9	21.8
Schwegel et al. 2024^28^	2.6	80^b^	80^b^	90	82	49^a^	54 ^a^	20	20	24	24	63	44	16	14
Sivamurugan et al. 2024^30^	5	77.7	77.9	79.5	80.1	N/R	N/R	41.4	40	N/R	N/R	N/R	N/R	N/R	N/R
Chi et al. 2024^29^	4.5	76.5	76.3	79	81.5	47.9	47.8	N/R	N/R	N/R	N/R	N/R	N/R	N/R	N/R

Abbreviations: C, control group; NYHA, New York Heart Association Functional Classification for Heart Failure; N/R, not reported; S, sodium‐glucose cotransporter 2 inhibitors.

^a^ Mean NYHA class.

^b^ Reported as median.

**FIGURE 2:**
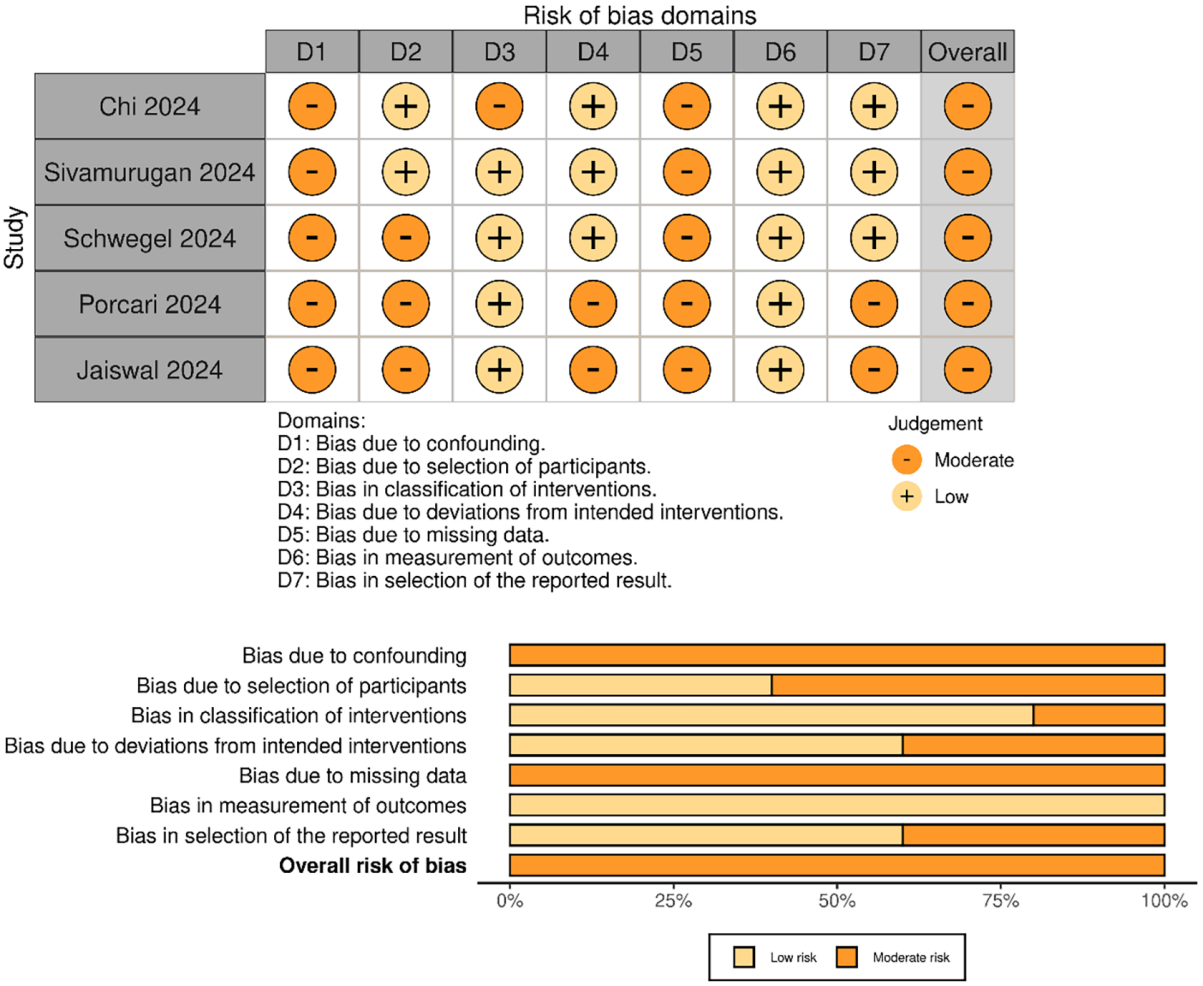
Risk of bias assessment based on the ROBINS‐I tool in a colorblind‐friendly colour scheme: Traffic‐light plot (top panel) and summary plot (bottom panel).

All studies were judged to have a moderate risk of bias, primarily due to their retrospective design and the potential for residual confounding, as assessed using the ROBINS‐I tool (Figure 2).

### 3.2 All‐cause mortality

**FIGURE 3:**
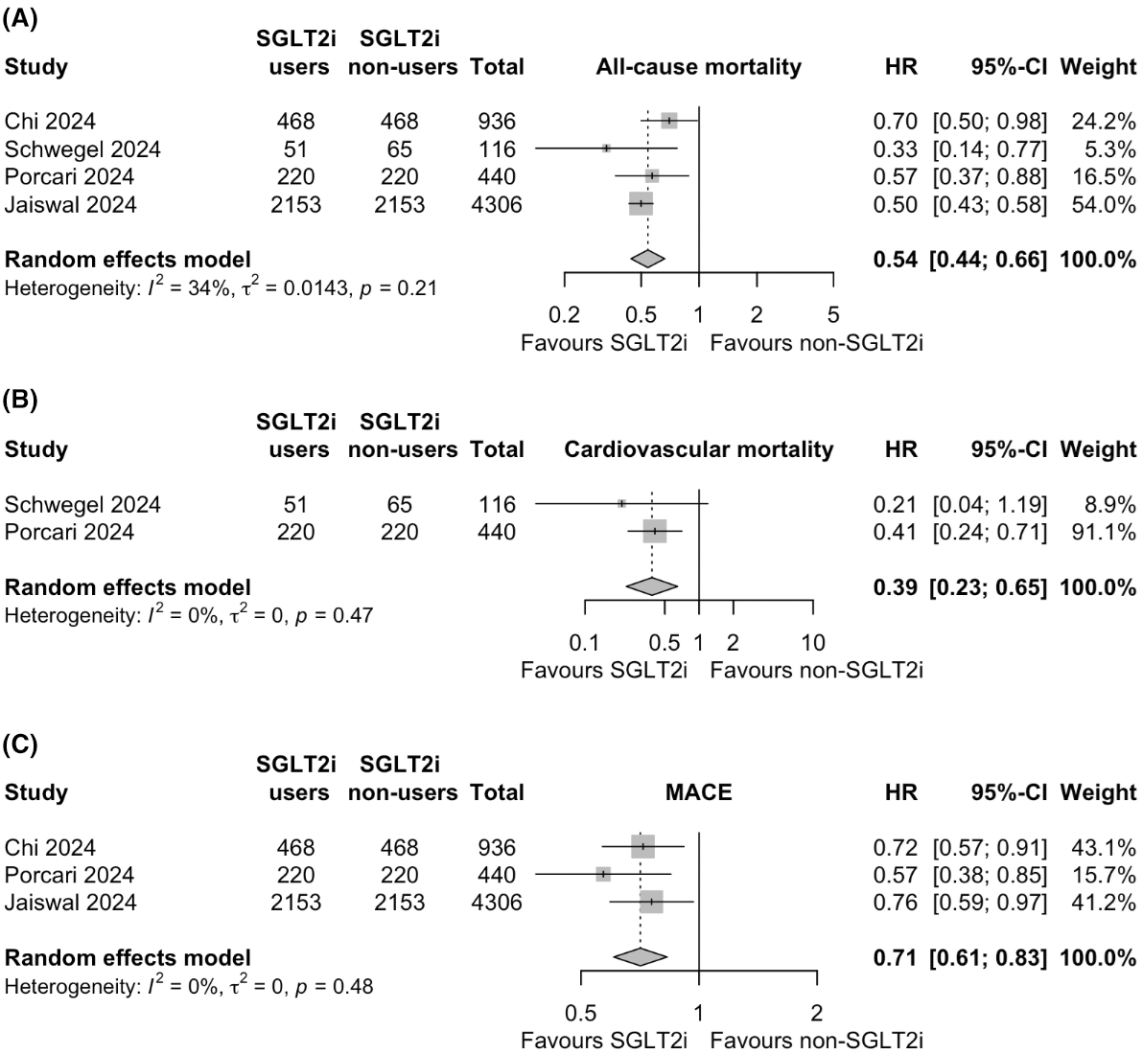
Forest plots of pairwise meta‐analyses on the effects of sodium‐glucose cotransporter 2 inhibitors (SGLT2i) on all‐cause mortality (A), cardiovascular mortality (B) and major adverse cardiovascular events (MACE, C). HR, hazard ratio; 95%‐CI, 95% confidence interval.

A total of 5 studies, including 5798 individuals, evaluated effect of SGLT2i on all‐cause mortality in patients with ATTR‐CM. The use of SGLT2i was significantly associated with a lower risk of all‐cause mortality compared to non‐use HR .54, 95% CI .44–.66, *p* < .001; *I* ^2^ = 35%, heterogeneity *p* = .21; (Figure 3A).

### 3.3 Cardiovascular mortality

Two studies assessed the impact of SGLT2i on cardiovascular mortality. SGLT2i users had a significantly lower risk of cardiovascular mortality compared to non‐users of SGLT2i (556 patients, HR .39, 95% CI .23–.65, *p* < .001; *I* ^2^ = 0%, heterogeneity *p* = .47; Figure 3B).

### 3.4 MACE

Three studies investigated the risk of MACE among SGLT2i users and non‐users. Based on the random effects meta‐analysis, the risk of MACE was 29% lower with SGLT2i treatment compared to no treatment (5682 patients, HR .71, 95% CI .61–.83, *p* < .001; *I* ^2^ = 0%, heterogeneity *p* = .48; Figure 3C).

### 3.5 HF hospitalization

**FIGURE 4:**
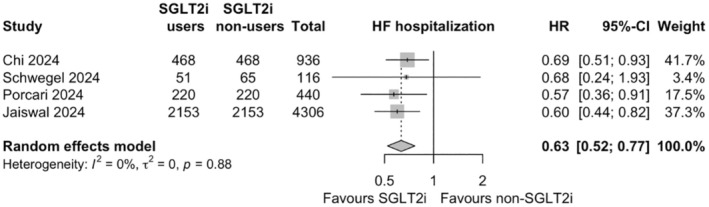
Forest plots of pairwise meta‐analyses on the effects of sodium‐glucose cotransporter 2 inhibitors (SGLT2i) on hospitalization for heart failure (HF). HR, hazard ratio; 95%‐CI, 95% confidence interval.

The risk of HHF was reported in 4 studies involving 4746 participants. SGLT2i use significantly reduced the risk of HHF compared to non‐use (HR .63, 95% CI .52–.77, *p* < .001; *I* ^2^ = 0%, heterogeneity *p* = .88; Figure 4).

### 3.6 Cardiac arrhythmias

**FIGURE 5:**
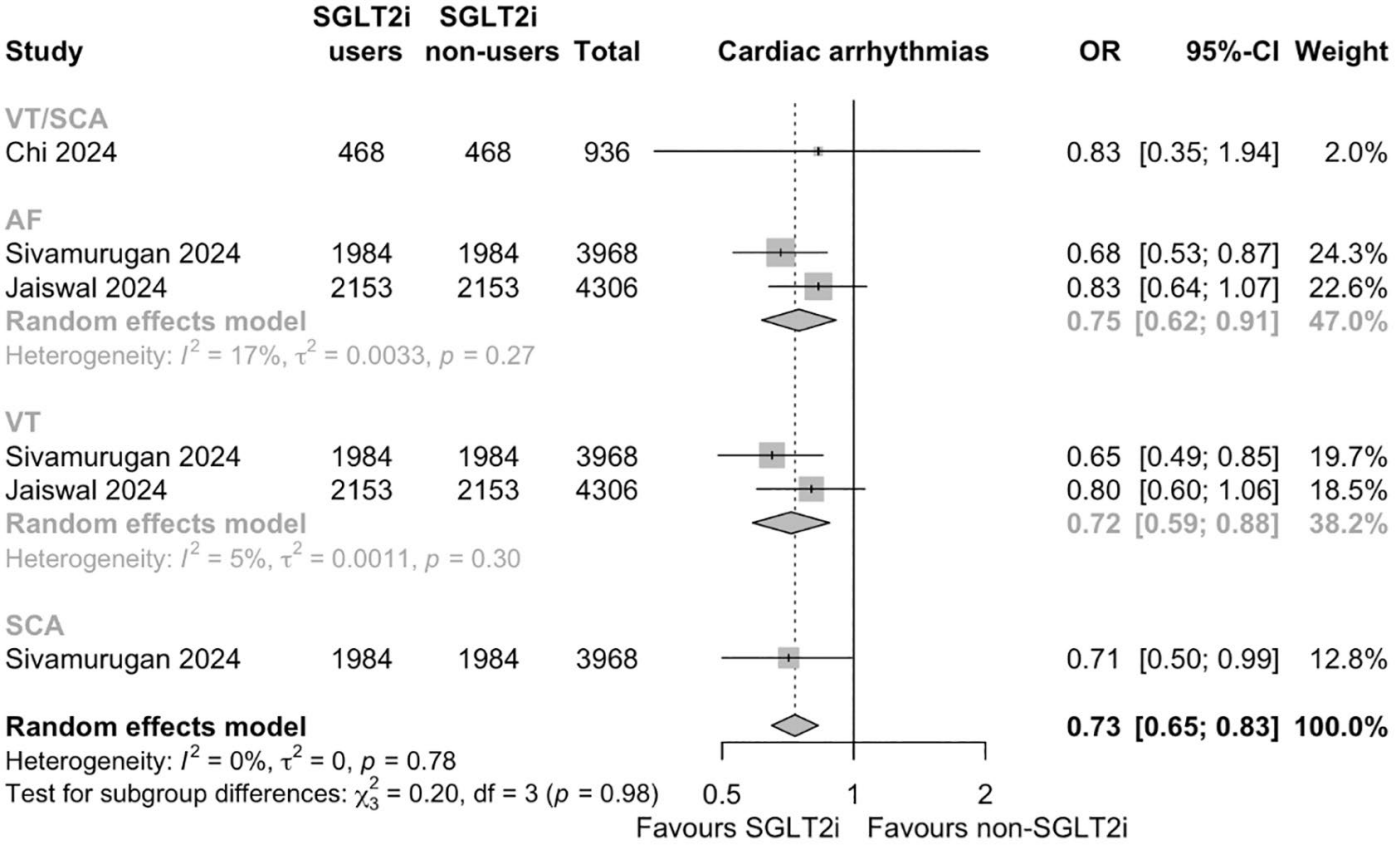
Forest plots of pairwise meta‐analyses on the effects of sodium‐glucose cotransporter 2 inhibitors (SGLT2i) on cardiac arrhythmias. AF, atrial fibrillation; OR, odds ratio; SCA, sudden cardiac death; VT, ventricular tachycardia; 95%‐CI, 95% confidence interval.

Three studies evaluated the impact of SGLT2i on cardiac arrhythmias involving 9210 ATTR‐CM patients. SGLT2i users had significantly lower odds of cardiac arrhythmias compared to non‐users (Odds ratio (OR) .73, 95% CI .65–.83, *p* < .001; *I* ^2^ = 0%, heterogeneity *p* = .78; Figure 5). Specifically, the use of SGLT2i was associated with a significant reduction in the odds of AF (8274 patients, OR .75, 95% CI .62–.91, *p* < .001; *I* ^2^ = 17%; Figure 5), VT (8274 patients, OR .72, 95% CI .59–.88, *p* < .001; *I* ^2^ = 5%; Figure 5) and SCA (3968 patients, OR .71, 95% CI .50–.99, *p* < .001; Figure 5).

### 3.7 Small study effects and publication bias

No evidence of small‐study effects (including publication bias) was detected, as indicated by symmetrical, contour‐enhanced funnel plots, suggesting minimal risk of bias in the pooled estimates (Figure S1).

## 4 DISCUSSION

This is the first meta‐analysis to demonstrate that the use of SGLT2i may be associated with an improved survival and more favourable outcomes in patients with ATTR‐CM. Specifically, SGLT2i users had a 46% lower risk of all‐cause mortality, a 61% lower risk of cardiovascular mortality, and a 29% lower risk of MACE compared to non‐users. Additionally, SGLT2i use was associated with a 37% reduction in the risk of HF‐related hospitalization and a 27% reduction in the odds of cardiac arrhythmias compared to non‐use. This reduction translated into significantly lower odds of AF, VT and/or SCA. The majority of patients had symptomatic HF, with approximately 10%–20% classified as NYHA class I. Hence, additional research is necessary to determine whether the observed benefits were primarily due to the established effects of SGLT2i on HF or their potential role as disease‐modifying agents in ATTR‐CM.

These findings align with previous analyses on the short‐term efficacy and safety of SGLT2i in patients with ATTR‐CM. In a retrospective cohort of 87 ATTR‐CM patients treated with SGLT2i, a median follow‐up of 6 months revealed significant improvements in the fluid status of these patients, demonstrated by reductions in weight, loop diuretic dose and uric acid levels.^17^ The authors also noted a transient decline in estimated glomerular filtration rate (eGFR) at 1 month post‐treatment initiation, though no sustained differences were observed in subsequent follow‐up.^17^ Cardiac biomarkers showed no significant between‐group differences, and SGLT2i was generally well tolerated, with a discontinuation rate of 11.5%.^17^

Similarly, a retrospective analysis evaluating the tolerability and short‐term clinical outcomes of dapagliflozin in 17 ATTR‐CM patients treated with tafamidis reported that 76.5% of dapagliflozin‐treated patients had a decrease in NT‐proBNP at 3 months follow‐up, with stable disease parameters and no adverse events reported.^18^ Another clinical feasibility study was conducted on 79 patients with ATTR‐CM who were treated with either dapagliflozin or empagliflozin.^31^ In this cohort, urinary tract infections occurred in 5.1% of patients, with two individuals discontinuing SGLT2i therapy, and 2.5% experiencing mortality unrelated to SGLT2i use.^31^ A mild decline in glomerular filtration rate was reported, while New York Heart Association (NYHA) functional status, cardiac and hepatic function and 6‐min walk distance remained stable over the study period, suggesting a potential role for SGLT2i in managing patients with amyloidosis and concurrent cardiac or renal dysfunction.^31^ These data, together with the findings of the present study, support the notion that SGLT2i represent a potentially safe and effective treatment option for patients with ATTR‐CM.

In a propensity score‐matched analysis of 220 patients with ATTR‐CM, Porcari et al. demonstrated significant benefits associated with SGLT2i therapy.^27^ Patients receiving SGLT2i showed a reduced likelihood of worsening NYHA functional class over 12 months compared to untreated patients.^27^ Additionally, SGLT2i treatment was linked to a slower progression of biomarker changes, including a reduced rate of NT‐proBNP increase and a slower decline in eGFR, after adjusting for baseline values.^27^ No significant differences in systolic blood pressure were observed between treated and untreated groups.^27^ Furthermore, SGLT2i therapy was associated with a marked reduction in the initiation of loop diuretics over 12 months among patients not requiring these agents at baseline.^27^

ATTR amyloidosis is strongly associated with renal impairment^32^ and albuminuria.^33^ Given the well‐documented link between kidney disease and an increased risk of adverse cardiovascular outcomes,^34^, ^35^, ^36^ the renoprotective effects of SGLT2i are particularly significant in reducing cardiovascular morbidity and mortality in patients with ATTR amyloidosis. Of note, a study involving 134 patients with ATTR‐CM revealed that the absence of SGLT2i therapy was a strong and independent predictor of kidney health, with SGLT2i use being associated with a 90% reduction in renal function decline.^37^ Hence, the reduction in mortality and morbidity observed in this meta‐analysis among ATTR‐CM patients receiving SGLT2i could, in part, be mediated through the preservation of renal function associated with SGLT2i therapy in this population.

Due to the structural and functional changes associated with transthyretin deposition (e.g., amyloid infiltration in the conduction system, atrial dilation secondary to diastolic dysfunction, etc.), arrhythmias are common in patients with ATTR‐CM. The management of these conditions is particularly challenging in this patient population due to high recurrence rates and the limited ability to fundamentally alter the disease course of ATTR‐CM.^38^, ^39^, ^40^, ^41^, ^42^ The present meta‐analysis suggests that SGLT2i confer significant benefits for ATTR‐CM, including a substantial reduction in the odds of arrhythmias, ranging from AF to VT and SCA. Notably, the magnitude of this reduction appears greater than that observed in the general population receiving SGLT2i treatment,^43^, ^44^ potentially due to the higher baseline arrhythmia risk in the ATTR‐CM population. Notably, a recent meta‐analysis of 33 RCTs involving over 48,000 patients receiving SGLT2i treatment demonstrated a significant 22% reduction in the risk of AF, which is slightly lower than the 25% reduction observed in the present analysis, along with a marginally non‐significant effect on the risk of SCA.^45^

The observed benefits of SGLT2i in the present study appear comparable to those reported in RCTs evaluating tafamidis and acoramidis for the endpoints of all‐cause mortality and HF hospitalizations.^6^, ^7^, ^8^ Notably, approximately 15%–20% of participants in our meta‐analysis were already receiving disease‐modifying treatments, suggesting that SGLT2i may confer additional benefit in this population. While the baseline characteristics of participants in our meta‐analysis were generally comparable to those in tafamidis and acoramidis trials with respect to age and diabetes prevalence, detailed comparisons for other variables, such as baseline chronic kidney disease were not possible due to a lack of pertinent data. It is also important to acknowledge inherent differences in the populations of the tafamidis and acoramidis trials, such as the percentage of patients with NYHA class II, which was 61.4% in the ATTR‐ACT trial (tafamidis) compared to 31.8% in the ATTRibute‐CM trial (acoramidis).^6^, ^7^, ^8^ These variations highlight the heterogeneity across studies and the need for cautious interpretation when comparing treatments.

### 4.1 Strengths and limitations

Several limitations should be considered when interpreting the findings of this meta‐analysis. Residual confounding remains a potential issue, as unmeasured factors may influence the results. The generalizability of these findings to other forms of cardiac amyloidosis is limited. Baseline data on key variables, including disease severity and the dosage of loop diuretics, were unavailable, potentially impacting the analysis. Although propensity score matching was employed in the primary studies to reduce confounding, it is well recognized that this statistical approach does not consistently replicate results observed in RCTs. The effect sizes observed in this study are larger than those reported in trials involving non‐amyloid populations, likely reflecting an overestimation due to methodological factors, particularly given the relatively low number of events in this dataset.

Despite propensity score matching, the possibility of unmeasured confounders cannot be entirely excluded, as this method does not fully account for all sources of bias. Furthermore, indication bias may have influenced SGLT2i treatment decisions, with some clinicians more inclined to prescribe these agents for patients with advanced disease, while others may have avoided them due to the lack of prior testing in ATTR‐CM populations. The inability to account for changes in clinical covariates between baseline and follow‐up, potentially linked to provider decisions, further limits the scope of the analysis.

This study was also underpowered for meaningful subgroup analyses, restricting our ability to draw conclusions across different patient subgroups. Although the investigators of the primary studies attempted to mitigate immortal time bias by initiating patient observation from the date of diagnosis rather than treatment initiation, this potential bias warrants consideration. Lastly, the findings of this analysis should be regarded as hypothesis‐generating. Confirmation through well‐designed, prospective, randomized, placebo‐controlled trials in contemporary ATTR‐CM cohorts receiving disease‐modifying therapies is essential to validate these results.

## 5 CONCLUSIONS

The use of SGLT2is may be associated with a more favourable prognosis in patients with ATTR‐CM. This benefit appears to translate into lower rates of all‐cause and cardiovascular mortality, MACE, HHF and cardiac arrhythmias, including AF, VT and SCA. Further research, particularly adequately powered, long‐term RCTs, is required to validate and strengthen the existing observational evidence.

## AUTHOR CONTRIBUTIONS

**Paschalis Karakasis**: conceptualization, methodology, investigation, visualization, project administration, writing—original draft, writing—review and editing. **Panagiotis Theofilis**: writing—review and editing. **Dimitrios Patoulias**: writing—review and editing. **Art Schuermans**: writing—review and editing. **Panayotis K Vlachakis**: writing—review and editing. **Aleksandra Klisic**: writing—review and editing. **Manfredi Rizzo**: writing—review and editing. **Nikolaos Fragakis**: conceptualization, methodology, investigation, writing—original draft, writing—review and editing. All authors read and approved the final manuscript.

## FUNDING INFORMATION

This research did not receive any specific grant from funding agencies in the public, commercial or not‐for‐profit sectors for its design or conduction.

## CONFLICT OF INTEREST STATEMENT

None declared.

## PROTOCOL

The protocol for this study is available at https://osf.io/5ft42/.

## Supporting information


Data S1:

## Data Availability

The data generated in this research will be shared on reasonable request to the corresponding author.
