# Supplementary material for: Sodium‐glucose cotransporter 2 inhibitors and outcomes in transthyretin amyloid cardiomyopathy: Systematic review and meta‐analysis
Source: Eur J Clin Invest. 2025 Jan 27;55(6):e14392. doi: 10.1111/eci.14392 (PMC12066899; doi:10.1111/eci.14392)
Supplement: Supplementary file 1 — Data S1: [file ECI-55-e14392-s001.docx]

**Supplementary material**

**Sodium-glucose cotransporter 2 inhibitors and outcomes in transthyretin amyloid cardiomyopathy: systematic review and meta-analysis**

Contents

[**Supplemental Table 1.** PRISMA 2020 Checklist 2](#_Toc184316310)

[**Supplemental Table 2.** Search strategy for MEDLINE (Pubmed) 4](#_Toc184316311)

[**Supplemental Table 3.** Search strategy for Cochrane library 5](#_Toc184316312)

[**Supplemental Table 4.** Search strategy for Scopus 5](#_Toc184316313)

[**Supplemental Table 5**. Variables used for confounder adjustment 6](#_Toc184316314)

[**Supplemental Figure 1**. Contour- enhanced funnel plot of effect size versus standard error for the association between SGLT2i use and all-cause mortality (A), major adverse cardiovascular events (B), heart failure hospitalization (C) and cardiac arrhythmias (D). 7](#_Toc184316315)

# **Supplemental Table 1.** PRISMA 2020 Checklist

| **Section and Topic** | **Item #** | **Checklist item** | **Location where item**  **is reported** |
| --- | --- | --- | --- |
| **TITLE** | | |  |
| Title | 1 | Identify the report as a systematic review. | 1 |
| **ABSTRACT** | | |  |
| Abstract | 2 | See the PRISMA 2020 for Abstracts checklist. | Done |
| **INTRODUCTION** | | |  |
| Rationale | 3 | Describe the rationale for the review in the context of existing knowledge. | 3 |
| Objectives | 4 | Provide an explicit statement of the objective(s) or question(s) the review addresses. | 3 |
| **METHODS** | | |  |
| Eligibility criteria | 5 | Specify the inclusion and exclusion criteria for the review and how studies were grouped for the syntheses. | 45 |
| Information sources | 6 | Specify all databases, registers, websites, organisations, reference lists and other sources searched or consulted to identify studies. Specify the date when each source was last searched or consulted. | 4 |
| Search strategy | 7 | Present the full search strategies for all databases, registers and websites, including any filters and limits used. | Sup. Tables 1-3 |
| Selection process | 8 | Specify the methods used to decide whether a study met the inclusion criteria of the review, including how many reviewers screened each record and each report retrieved, whether they worked independently, and if applicable, details of automation tools used in the process. | 5 |
| Data collection process | 9 | Specify the methods used to collect data from reports, including how many reviewers collected data from each report, whether they worked independently, any processes for obtaining or confirming data from study investigators, and if applicable, details of automation tools used in the process. | 5 |
| Data items | 10a | List and define all outcomes for which data were sought. Specify whether all results that were compatible with each outcome domain in each study were sought (e.g. for all measures, time points, analyses), and if not, the methods used to decide which results to collect. | 4 |
|  | 10b | List and define all other variables for which data were sought (e.g. participant and intervention characteristics, funding sources). Describe any assumptions made about any missing or unclear information. | 5 |
| Study risk of bias assessment | 11 | Specify the methods used to assess risk of bias in the included studies, including details of the tool(s) used, how many reviewers assessed each study and whether they worked independently, and if applicable, details of automation tools used in the process. | 5 |
| Effect measures | 12 | Specify for each outcome the effect measure(s) (e.g. risk ratio, mean difference) used in the synthesis or presentation of results. | 5-6 |
| Synthesis methods | 13a | Describe the processes used to decide which studies were eligible for each synthesis (e.g. tabulating the study intervention characteristics and comparing against the planned groups for each synthesis (item #5)). | 5-6 |
|  | 13b | Describe any methods required to prepare the data for presentation or synthesis, such as handling of missing summary statistics, or data conversions. | 5-6 |
|  | 13c | Describe any methods used to tabulate or visually display results of individual studies and syntheses. | 5-6 |
|  | 13d | Describe any methods used to synthesize results and provide a rationale for the choice(s). If meta-analysis was performed, describe the model(s), method(s) to identify the presence and extent of statistical heterogeneity, and software package(s) used. | 5-6 |
|  | 13e | Describe any methods used to explore possible causes of heterogeneity among study results (e.g. subgroup analysis, meta-regression). | 5-6 |
|  | 13f | Describe any sensitivity analyses conducted to assess robustness of the synthesized results. | 5-6 |
| Reporting bias assessment | 14 | Describe any methods used to assess risk of bias due to missing results in a synthesis (arising from reporting biases). | 5-6 |
| Certainty assessment | 15 | Describe any methods used to assess certainty (or confidence) in the body of evidence for an outcome. | NA |
| **Section and Topic** | **Item #** | **Checklist item** | **Location where item**  **is reported** |
| **RESULTS** | | |  |
| Study selection | 16a | Describe the results of the search and selection process, from the number of records identified in the search to the number of studies included in the review, ideally using a flow diagram. | Sup. Fig. 1 |
|  | 16b | Cite studies that might appear to meet the inclusion criteria, but which were excluded, and explain why they were excluded. | Sup. Table 4 |
| Study characteristics | 17 | Cite each included study and present its characteristics. | 6-7 and Table 1 |
| Risk of bias in studies | 18 | Present assessments of risk of bias for each included study. | Sup. Table 5 |
| Results of individual studies | 19 | For all outcomes, present, for each study: (a) summary statistics for each group (where appropriate) and (b) an effect estimate and its precision (e.g. confidence/credible interval), ideally using structured tables or plots. | Fig. 1-4 |
| Results of syntheses | 20a | For each synthesis, briefly summarise the characteristics and risk of bias among contributing studies. | 7-8 |
|  | 20b | Present results of all statistical syntheses conducted. If meta-analysis was done, present for each the summary estimate and its precision (e.g. confidence/credible interval) and measures of statistical heterogeneity. If comparing groups, describe the direction of the effect. | 7-8 |
|  | 20c | Present results of all investigations of possible causes of heterogeneity among study results. | 7-8 |
|  | 20d | Present results of all sensitivity analyses conducted to assess the robustness of the synthesized results. | 7-8 |
| Reporting biases | 21 | Present assessments of risk of bias due to missing results (arising from reporting biases) for each synthesis assessed. | 8, Sup. Fig. 3 |
| Certainty of evidence | 22 | Present assessments of certainty (or confidence) in the body of evidence for each outcome assessed. | NA |
| **DISCUSSION** | | |  |
| Discussion | 23a | Provide a general interpretation of the results in the context of other evidence. | 8-11 |
|  | 23b | Discuss any limitations of the evidence included in the review. | 11 |
|  | 23c | Discuss any limitations of the review processes used. | 11 |
|  | 23d | Discuss implications of the results for practice, policy, and future research. | 8-12 |
| **OTHER INFORMATION** | | |  |
| Registration and protocol | 24a | Provide registration information for the review, including register name and registration number, or state that the review was not registered. | 3 |
|  | 24b | Indicate where the review protocol can be accessed, or state that a protocol was not prepared. | 3, 12 |
|  | 24c | Describe and explain any amendments to information provided at registration or in the protocol. | NA |
| Support | 25 | Describe sources of financial or non-financial support for the review, and the role of the funders or sponsors in the review. | 12 |
| Competing interests | 26 | Declare any competing interests of review authors. | 12 |
| Availability of data, code and other materials | 27 | Report which of the following are publicly available and where they can be found: template data collection forms; data extracted from included studies; data used for all analyses; analytic code; any other materials used in the review. | 12 |

*From:* Page MJ, McKenzie JE, Bossuyt PM, Boutron I, Hoffmann TC, Mulrow CD, et al. The PRISMA 2020 statement: an updated guideline for reporting systematic reviews. BMJ 2021;372:n71. doi: 10.1136/bmj.n71

For more information, visit: <http://www.prisma-statement.org/>

# **Supplemental Table 2.** Search strategy for MEDLINE (Pubmed)

| (("sodium-glucose"[All Fields] AND "co-transporter-2"[All Fields] AND "inhibitor*"[All Fields]) OR "sodium glucose transporter 2 inhibitors"[MeSH Terms] OR ("SGLT-2"[All Fields] AND "inhibitor*"[All Fields]) OR (("sodium glucose transport proteins"[MeSH Terms] OR ("sodium-glucose"[All Fields] AND "transport"[All Fields] AND "proteins"[All Fields]) OR "sodium glucose transport proteins"[All Fields] OR ("sodium"[All Fields] AND "glucose"[All Fields] AND "cotransporter"[All Fields]) OR "sodium glucose cotransporter"[All Fields]) AND "2"[All Fields]) OR ("SGLT2"[All Fields] AND "inhibitor*"[All Fields]) OR (("sodium glucose transport proteins"[MeSH Terms] OR ("sodium-glucose"[All Fields] AND "transport"[All Fields] AND "proteins"[All Fields]) OR "sodium glucose transport proteins"[All Fields] OR ("sodium"[All Fields] AND "glucose"[All Fields] AND "cotransporter"[All Fields]) OR "sodium glucose cotransporter"[All Fields]) AND "2"[All Fields] AND "inhibitor*"[All Fields]) OR ("empagliflozin"[Supplementary Concept] OR "empagliflozin"[All Fields]) OR ("dapagliflozin"[Supplementary Concept] OR "dapagliflozin"[All Fields] OR "dapagliflozin s"[All Fields]) OR ("canagliflozin"[MeSH Terms] OR "canagliflozin"[All Fields]) OR ("2s 3r 4r 5s 6r 2 4 chloro 3 4 ethoxybenzyl phenyl 6 methylthio tetrahydro 2h pyran 3 4 5 triol"[Supplementary Concept] OR "2s 3r 4r 5s 6r 2 4 chloro 3 4 ethoxybenzyl phenyl 6 methylthio tetrahydro 2h pyran 3 4 5 triol"[All Fields] OR "sotagliflozin"[All Fields]) OR ("ertugliflozin"[Supplementary Concept] OR "ertugliflozin"[All Fields]) OR ("bexagliflozin"[Supplementary Concept] OR "bexagliflozin"[All Fields]) OR ("ipragliflozin"[Supplementary Concept] OR "ipragliflozin"[All Fields]) OR ("6 4 ethylphenyl methyl 3 4 5 6 tetrahydro 6 hydroxymethyl spiro isobenzofuran 1 3h 2 2h pyran 3 4 5 triol"[Supplementary Concept] OR "6 4 ethylphenyl methyl 3 4 5 6 tetrahydro 6 hydroxymethyl spiro isobenzofuran 1 3h 2 2h pyran 3 4 5 triol"[All Fields] OR "tofogliflozin"[All Fields]) OR ("1 5 anhydro 1 5 4 ethoxybenzyl 2 methoxy 4 methylphenyl 1 thioglucitol"[Supplementary Concept] OR "1 5 anhydro 1 5 4 ethoxybenzyl 2 methoxy 4 methylphenyl 1 thioglucitol"[All Fields] OR "luseogliflozin"[All Fields]) OR ("licogliflozin"[Supplementary Concept] OR "licogliflozin"[All Fields]) OR ("sergliflozin"[Supplementary Concept] OR "sergliflozin"[All Fields]) OR "remogliflozin"[All Fields]) |
| --- |
| AND |
| ("transthyretin amyloid cardiomyopathy"[Title/Abstract] OR "transthyretin"[Title/Abstract]) |

# **Supplemental Table 3.** Search strategy for Cochrane library

| #1 | MeSH descriptor: [Sodium-Glucose Transporter 2 Inhibitors] explode all trees |
| --- | --- |
| #2 | sglt2 inhibitor |
| #3 | empagliflozin |
| #4 | dapagliflozin |
| #5 | canagliflozin |
| #6 | sotagliflozin |
| #7 | ertugliflozin |
| #8 | bexagliflozin |
| #9 | ipragliflozin |
| #10 | tofogliflozin |
| #11 | luseogliflozin |
| #12 | licogliflozin |
| #13 | sergliflozin |
| #14 | remogliflozin |
| #15 | transthyretin amyloid cardiomyopathy |
| #16 | transthyretin |
| #17 | amyloid cardiomyopathy |
| #18 | #1 OR #2 OR #3 OR #4 OR #5 OR #6 OR #7 OR #8 OR #9 OR #10 OR #11 OR #12 OR #13 OR #14 |
| #19 | #15 OR #16 OR #17 |
| #20 | #18 AND #19 |

# **Supplemental Table 4.** Search strategy for Scopus

| ( ( sodium-glucose AND co-transporter-2 AND inhibitor ) OR ( sodium-glucose AND transporter 2 inhibitors ) OR ( sglt-2 AND inhibitor ) OR ( sodium-glucose AND cotransporter 2 ) OR ( sglt2 AND inhibitor ) OR ( sodium-glucose AND cotransporter 2 inhibitor ) OR ( empagliflozin ) OR ( dapagliflozin ) OR ( canagliflozin ) OR ( sotagliflozin ) OR ( ertugliflozin ) OR ( bexagliflozin ) OR ( ipragliflozin ) OR ( tofogliflozin ) OR ( luseogliflozin ) OR ( licogliflozin ) OR ( sergliflozin ) OR ( remogliflozin ) ) |
| --- |
| AND |
| ( TITLE-ABS ( transthyretin AND amyloid AND cardiomyopathy ) OR TITLE-ABS ( transthyretin ) ) |

# **Supplemental Table 5**. Variables used for confounder adjustment

| **Reference** | **Variables** |
| --- | --- |
| Porcari A, Cappelli F, Nitsche C, Tomasoni D, Sinigiani G, Longhi S, et al. SGLT2 Inhibitor Therapy in Patients With Transthyretin Amyloid Cardiomyopathy. J Am Coll Cardiol 2024;83:2411–22. https://doi.org/10.1016/j.jacc.2024.03.429 | Year of diagnosis, sex, diabetes mellitus, hypertension, atrial fibrillation, estimated glomerular filtration rate, wild-type or hereditary ATTR-CM, maximum wall thickness in diastole, left ventricular ejection fraction (LVEF), treatment with beta-blockers, treatment with angiotensin-converting enzyme inhibitors (ACEI) or angiotensin receptor blockers (ARB), treatment with MRA, treatment with loop diuretic agents, and treatment with disease-modifying drugs, age and N-terminal pro–B-type natriuretic peptide (NT-proBNP). |
| Schwegel N, Toferer C, Zach DK, Santner V, Höller V, Lugitsch J, et al. Impact of SGLT2-Inhibitor Therapy on Survival in Patients with Transthyretin Amyloid Cardiomyopathy: Analysis of a Prospective Registry Study. J Clin Med 2024;13. https://doi.org/10.3390/jcm13195966 | Estimated glomerular filtration rate (eGFR), NT-proBNP, LVEF, and concomitant therapy with the selective transthyretin stabilizer tafamidis. |
| Jaiswal V, Hanif M, Mashkoor Y, Jaiswal A, Prasad T, Rajak K, et al. Sodium-Glucose Cotransporter 2 Inhibitor Use and Outcomes in Transthyretin Amyloid Cardiomyopathy. JACC Adv 2024:101405. https://doi.org/10.1016/J.JACADV.2024.101405 | Age, sex, race, body mass index, hypertension, diabetes mellitus, chronic kidney disease, smoking status, hemoglobin level, low-density lipid level, left ventricular ejection fraction, New York Heart Association (NYHA) class of heart failure, and various drugs including tafamidis, angiotensin-converting enzyme inhibitors, angiotensin receptor blockers, angiotensin receptor/neprilysin inhibitor, beta-blockers, diuretics, spironolactone, eplerenone, and statins. |
| Sivamurugan A, Byer SH, Grewal US, Dominic P. Impact of SGLT2 Inhibitors on the Outcomes of Patients with Cardiac Arrythmias and Transthyretin Cardiac Amyloidosis. Hear Rhythm 2024;0. https://doi.org/10.1016/j.hrthm.2024.09.002 | Age, race, sex and co-morbidities. |
| Chi KY, Borkowski P, Osabutey A, Varrias D, Song J, Chiang CH, et al. Abstract 4142228: The Association Between Sodium-glucose Cotransporter-2 Inhibitors and Major Adverse Cardiac Events in Tafamidis-treated Transthyretin Amyloid Cardiomyopathy: A Real-World Analysis. Circulation 2024;150. https://doi.org/10.1161/CIRC.150.SUPPL_1.4142228 | Important covariates were also balanced between two arms. All patients were on tafamidis treatment. |


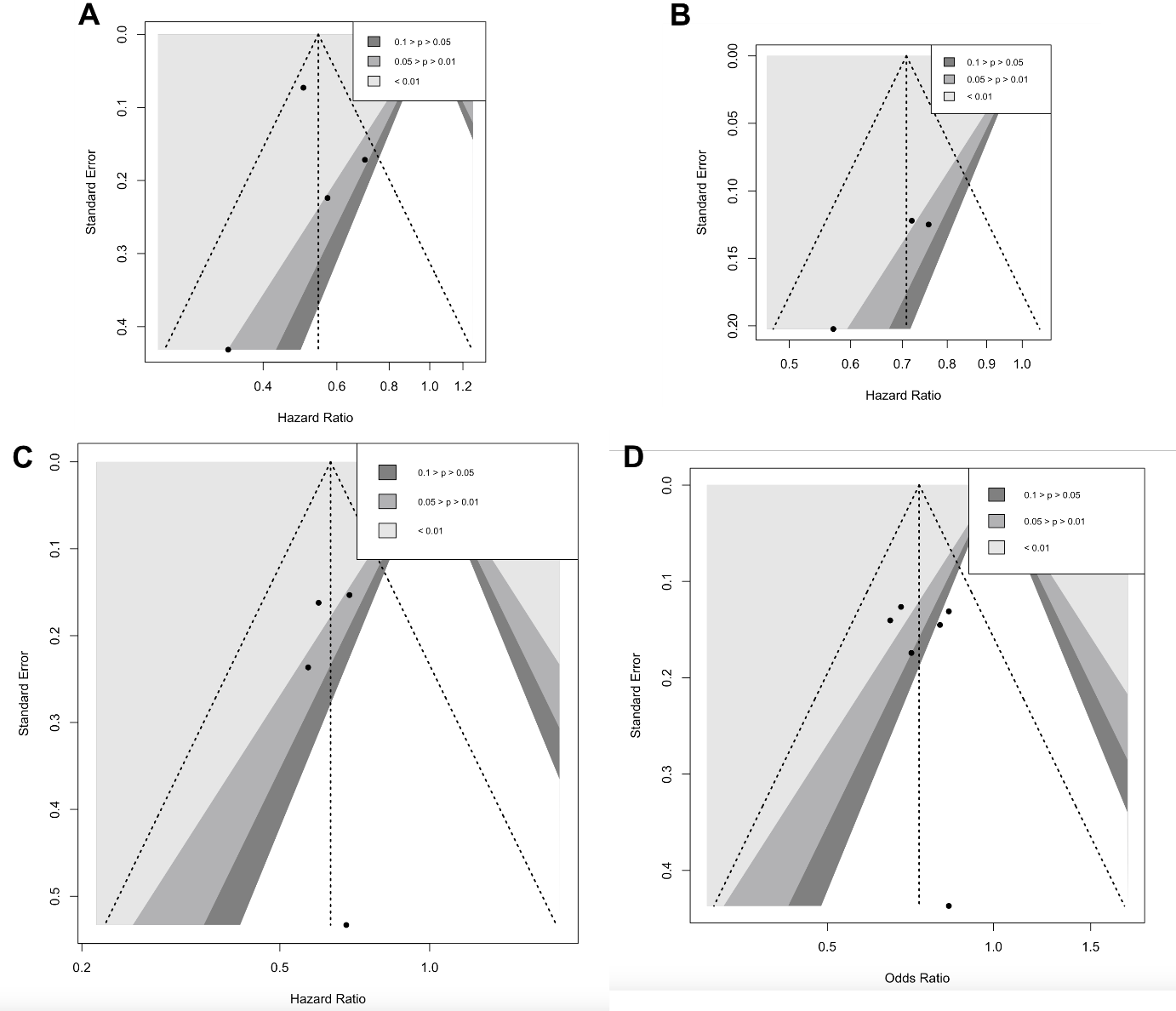


# **Supplemental Figure 1**. Contour- enhanced funnel plot of effect size versus standard error for the association between SGLT2i use and all-cause mortality (A), major adverse cardiovascular events (B), heart failure hospitalization (C) and cardiac arrhythmias (D).
